# Comprehensive bioinformatics analysis of hub genes in ischemic heart failure and atrial fibrillation

**DOI:** 10.3389/fcvm.2025.1499065

**Published:** 2025-02-24

**Authors:** Meimei Zhou, Youkang Xu, Lili Zhang, Yushan Yang, Jiejiao Zheng

**Affiliations:** ^1^ Department of Rehabilitation, Huadong Hospital, Fudan University, Shanghai, China; ^2^ Department of Osteoarthropathy Rehabilitation, The Second Rehabilitation Hospital of Shanghai, Shanghai, China

**Keywords:** heart failure, atrial fibrillation, common differentially expressed genes, biomarkers, hub genes, ischemic heart failure

## Abstract

**Background:**

Atrial fibrillation (AF) and heart failure (HF) frequently coexist and mutually influence each other. The association between AF and the subtype of HF, Ischaemic heart failure (IHF), remains insufficiently described, despite their high prevalence. Hence, comprehending their underlying pathophysiological mechanisms and identifying new therapeutic targets are urgently needed.

**Objective:**

This exploration aims to unearth related genes and pathways of IHF and AF, offering new perspectives for their joint diagnosis and treatment.

**Methods:**

Datasets for HF (GSE57338) and AF (GSE128188) were acquired from the Gene Expression Omnibus (GEO) database. Intersecting these sets generated common differentially expressed genes (DEGs) for further analyses, including Gene Ontology (GO) enrichment, Kyoto Encyclopedia of Genes and Genomes (KEGG) pathways, protein-protein interaction (PPI), and hub gene identification. Subsequently, the HF dataset (GSE116250) and AF dataset (GSE2240) were utilized to confirm the expression of the hub genes, followed by examination of gene expression patterns across cells in single-cell datasets.

**Results:**

The study identified 20 common DEGs. Among them, 10 hub genes (SFRP4, FMOD, HAPLN1, LTBP2, SVEP1, BCL6, ANPEP, CD38, ATRNL1, and BEX1) were found to be associated with the co-occurrence of IHF and AF. Enrichment analysis revealed the predominant involvement of these hub genes in extracellular matrix (ECM). Data from the Uniprot database revealed the involvement of the Wnt signaling pathway and TGF-β1/Smads signaling pathway in the development and progression of AF and IHF. Single-cell analysis demonstrated high gene expression primarily in monocytes.

**Conclusion:**

The identified 10 hub genes can serve as potentially valuable biomarkers for IHF and AF. Enrichment analysis reveals that these potential biomarkers are significantly associated with ECM, nicotinate, and nicotinamide metabolism, providing a foundational target for the joint diagnosis and treatment of the two diseases.

## 1 Introduction

Atrial fibrillation (AF) and heart failure (HF) rank among the most prevalent cardiovascular diseases, with their global incidence continually escalating (1, 2). AF and HF share numerous risk factors such as age, obesity, hypertension, heart valve disease, ischemic heart disease, and thyroid dysfunction, contributing to their co-occurrence. Approximately 10%–50% of end-stage HF patients manifest concurrent AF, exacerbating each other in a detrimental cycle (3, 4). AF can worsen HF patients' heart function, accelerate symptom onset, and impair quality of life, just as HF can intensify AF.

Two primary HF subtypes are ischemic heart disease (ISCH) and dilated cardiomyopathy (DCM) (5). Despite sharing similar symptoms, the two subtypes may generate distinct structural or functional phenotypes, influencing treatment response (5–7). ISCH patients generally face a reduced survival rate compared to DCM patients (5, 6), and AF seems to elevate death risk exclusively in ISCH patients (8, 9). Catheter ablation, a treatment for AF in HF, shows limited efficacy and safety, with a high risk of recurrence and significant costs (10). These challenges necessitate optimized treatment strategies for AF in HF patients. A thorough understanding of molecular mechanisms and pathogenesis between these conditions is essential for enhancing early diagnosis, treatment efficacy, and patient prognosis.

Current research into the mechanism of AF and HF has not sufficiently explored the bidirectional association between AF and IHF, particularly in bioinformatics-based genetic mechanisms. Bioinformatics analysis of microarray gene expression profiles offers novel insights into molecular mechanisms underlying both diseases. However, studies reporting interactions between differentially expressed genes (DEGs) and key signaling pathway genes in HF and AF remain limited (11, 12). This study analyzed public datasets and identified hub genes through protein-protein interaction network analysis to investigate the biological mechanisms of IHF and AF, aiming to reveal related genes and pathways for improved joint diagnosis and treatment approaches.

## 2 Materials and methods

### 2.1 Data sources

The Gene Expression Omnibus (GEO) database (https://www.ncbi.nlm.nih.gov/geo/) was searched using keywords “atrial fibrillation,” “heart failure,” and “RNA-seq” to identify relevant datasets. The search yielded HF datasets (GSE57338, GSE116250) and AF datasets (GSE128188, GSE2240). In the GSE57338 dataset, the GPL11532 [HuGene-1_1-st] Affymetrix Human Gene 1.1 ST Array [transcript (gene) version] was used, comprising 95 ISCH patients and 136 non-HF individuals. Likewise, GSE116250 [GPL16791 Illumina HiSeq 2,500 (Homo sapiens)] encompasses 13 ISCH patients and 14 non-HF donors, while GSE128188 [GPL18573 Illumina NextSeq 500 (Homo sapiens)] includes 5 patients with sinus rhythm (SR) and 5 patients with AF. GSE2240 [GPL97 (HG-U133B) Affymetrix Human Genome U133B Array] contains 20 patients with SR and 10 patients with permanent AF.

Due to limited PBMC sequencing data availability in HF samples, PBMC sequencing data were obtained from GSM8271496 and GSM8271498 samples. The GSM8271498 sample contained 11,421 genes and 90,483 cells, while GSM8271468 included 10,884 genes and 90,483 cells.

### 2.2 Identification of DEGs

DEGs were identified using R software (version 4.2.3) with two specialized packages: “limma” (version 3.54.2) and “edgeR” (version 3.40.2). These packages enabled differential expression analyses for RNA-sequencing and microarray studies. DEGs were selected from the HF dataset (GSE57338) and AF dataset (GSE128188) using criteria of |Log2 Fold Change| > 0.6 and |adj.P.Val.| < 0.05. The “ggplot2” package (version 3.3.6) generated volcano plots for DEG visualization.

### 2.3 Screening of common DEGs

To identify the common jointly up- and down-regulated DEGs of HF and AF, the “vennDiagram” package (version 1.7.3) in R software was employed, and these common DEGs were visualized in a Venn diagram. Subsequent analysis was then conducted on these common DEGs.

### 2.4 Functional enrichment analysis

Subsequently, gene ontology (GO) analysis and Kyoto encyclopedia of gene and genomes (KEGG) pathway enrichment analysis were executed on common DEGs screened from HF patients and AF patients. The “clusterProfiler” package (version 4.6.2) in R software facilitated the exploration of biological functions and related pathways through comparison of biological themes among gene clusters. A *P* < 0.05 was considered statistically significant in GO and KEGG analyses. GO analysis was subdivided into three components: biological process (BP), cellular component (CC), and molecular function (MF).

### 2.5 Protein-protein interaction network construction and hub gene analysis

Protein-protein interaction (PPI) networks were constructed using the Search Tool for the Retrieval of Interacting Genes/Proteins (STRING) online platform (https://www.string-db.org) for common DEGs, considering interaction scores >0.15 as significant. The corresponding results were downloaded and saved locally for subsequent visualization, which was accomplished through Cytoscape V3.9.1 software. Within this visualization, the key top 10 genes were identified using the cytoHubba plugin, and subnetworks were found with MCODE. Furthermore, a PPI network analysis of these top 10 key genes was conducted using GeneMANIA to explore the potential interactions among them.

The Uniprot database (https://www.uniprot.org/), which provides protein sequences, functional information, and research paper references, integrates resources from the European Bioinformatics Institute (EBI), the Swiss Institute of Bioinformatics (SIB), and the Protein Information Resource (PIR). This database was utilized to explore the functional pathways of the 10 identified genes, aiming to uncover potential mechanisms associated with the development and progression of AF and IHF.

### 2.6 Validation of hub gene expression

The expression of the selected hub genes was confirmed using the GSE116250 and GSE2240 datasets. A *P* < 0.05 was considered significant.

### 2.7 Single-cell analysis of hub gene locations

The expression of 10 hub genes in the HF microenvironment was analyzed using single-cell transcriptomic datasets GSM8271496 and GSM8271498. Single-cell transcriptome data preprocessing employed the “Seurat” package (version 5.0.3). Quality control parameters included cell filtering with nFeature_RNA thresholds (200-8000) and percent.MT below 25. Data normalization and variance stabilization preceded the analysis of 2,000 highly variable genes per sample. Dimensional reduction utilized the ScaleData and RunPCA functions sequentially. Cell clustering implemented FindNeighbors and FindClusters functions. The UMAP method facilitated cell annotation and differential gene expression analysis across cell populations.

## 3 Results

### 3.1 Identification of DEGs in HF and AF datasets

**Figure 1:**
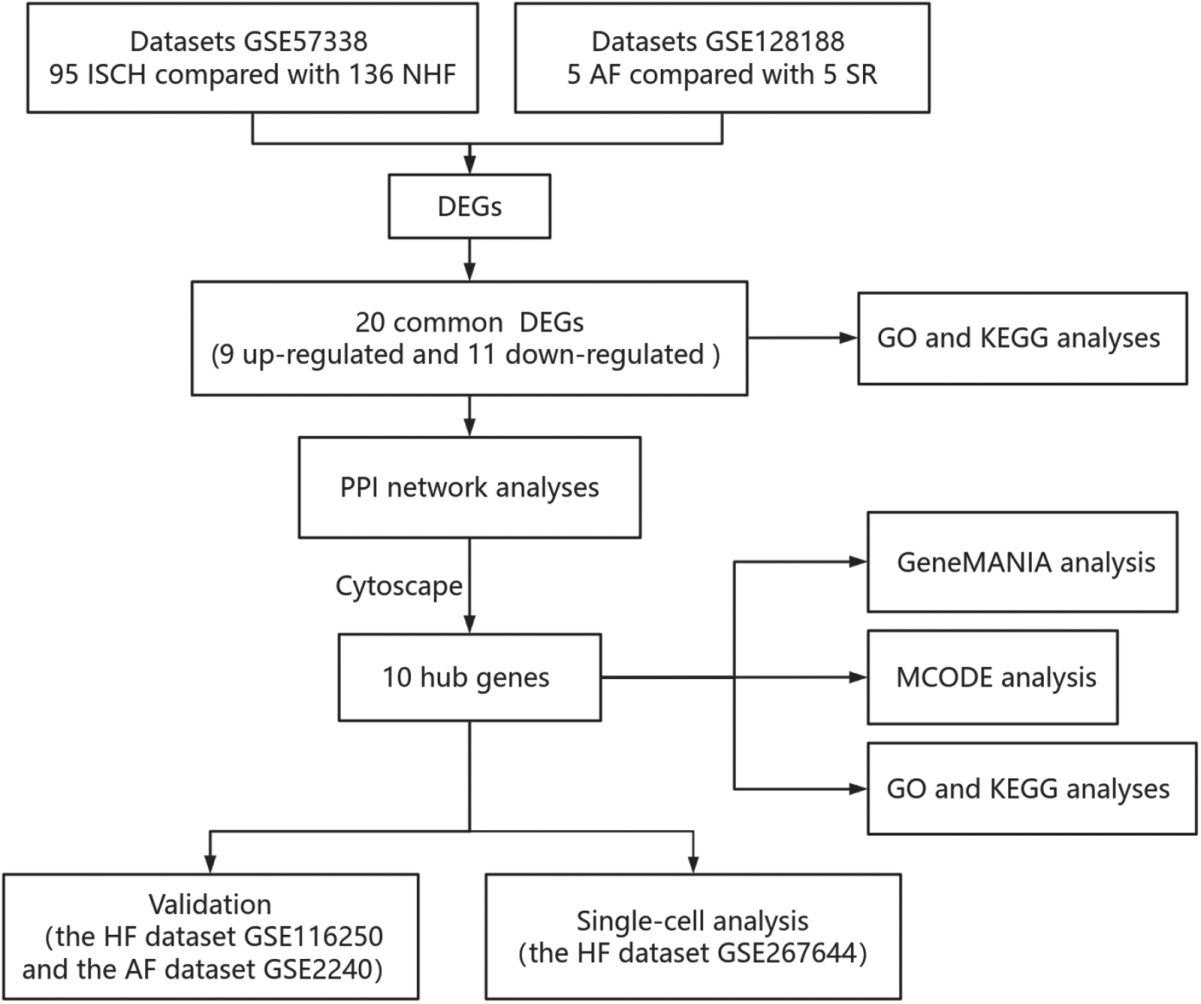
Research design flowchart.

**Figure 2:**
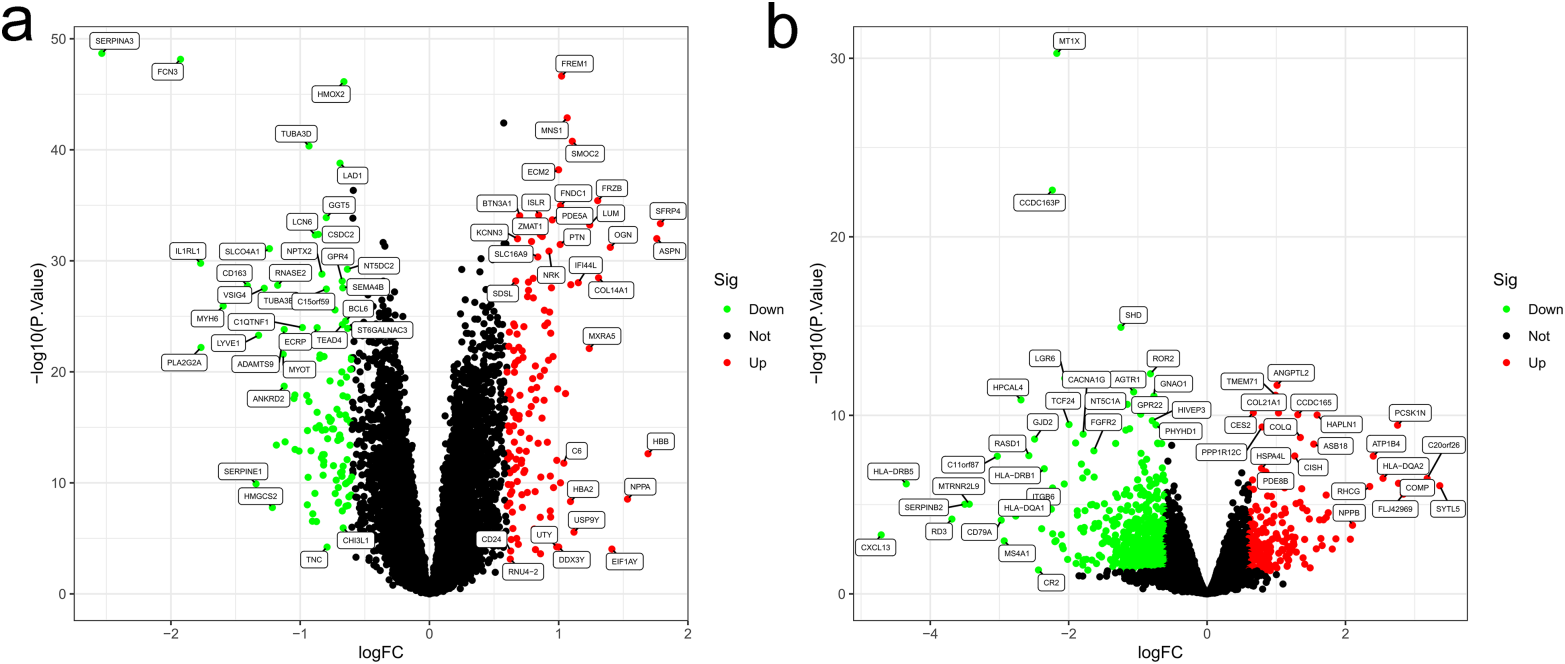
Volcano plots. **(a)** Volcano plot of GSE57338; **(b)** volcano plot of GSE128188. Up-regulated genes are highlighted in light red, and down-regulated genes are highlighted in light green.

From the GEO database, GSE57338 (comprising 95 ISCH patients and 136 non-HF individuals) and GSE128188 (containing 5 permanent AF patients and 5 SR patients) were downloaded (Figure 1). In the GSE57338 dataset, we identified 246 DEGs, with 136 up-regulated and 110 down-regulated. Similarly, in the GSE128188 dataset, we found 365 DEGs, of which 107 were up-regulated and 258 were down-regulated. These results are illustrated in the volcano plot (Figures 2a,b).

### 3.2 Screening of common up- and down-regulated DEGs in HF and AF datasets

**Figure 3:**
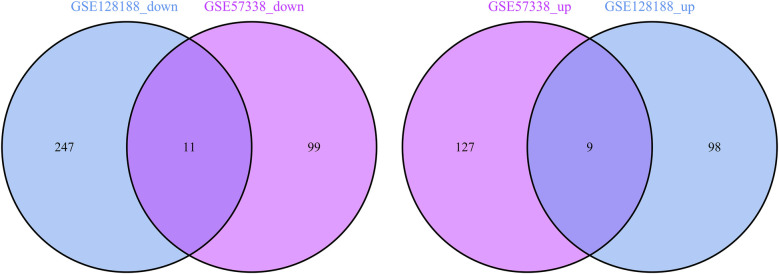
Venn diagram of common down-regulated genes and common up-regulated DEGs.

A meticulous co-expression analysis was conducted on up- and down-regulated DEGs in the datasets GSE57338 and GSE128188, respectively. The results yielded 9 common up-regulated DEGs (TMEM71, HAPLN1, ATRNL1, LTBP2, FMOD, SFRP4, BEX1, SVEP1, PHLDA1) and 11 common down-regulated DEGs (MT1X, BCL6, CSDC2, C1orf105, CD38, FAM46B, AREG, FAM83B, ANPEP, MT1A, METTL7B) (Figure 3). These findings demonstrated significant differential gene expression patterns between IHF and AF patients, suggesting potential roles in disease progression.

### 3.3 Enrichment pathways and analysis for common DEGs

**Figure 4:**
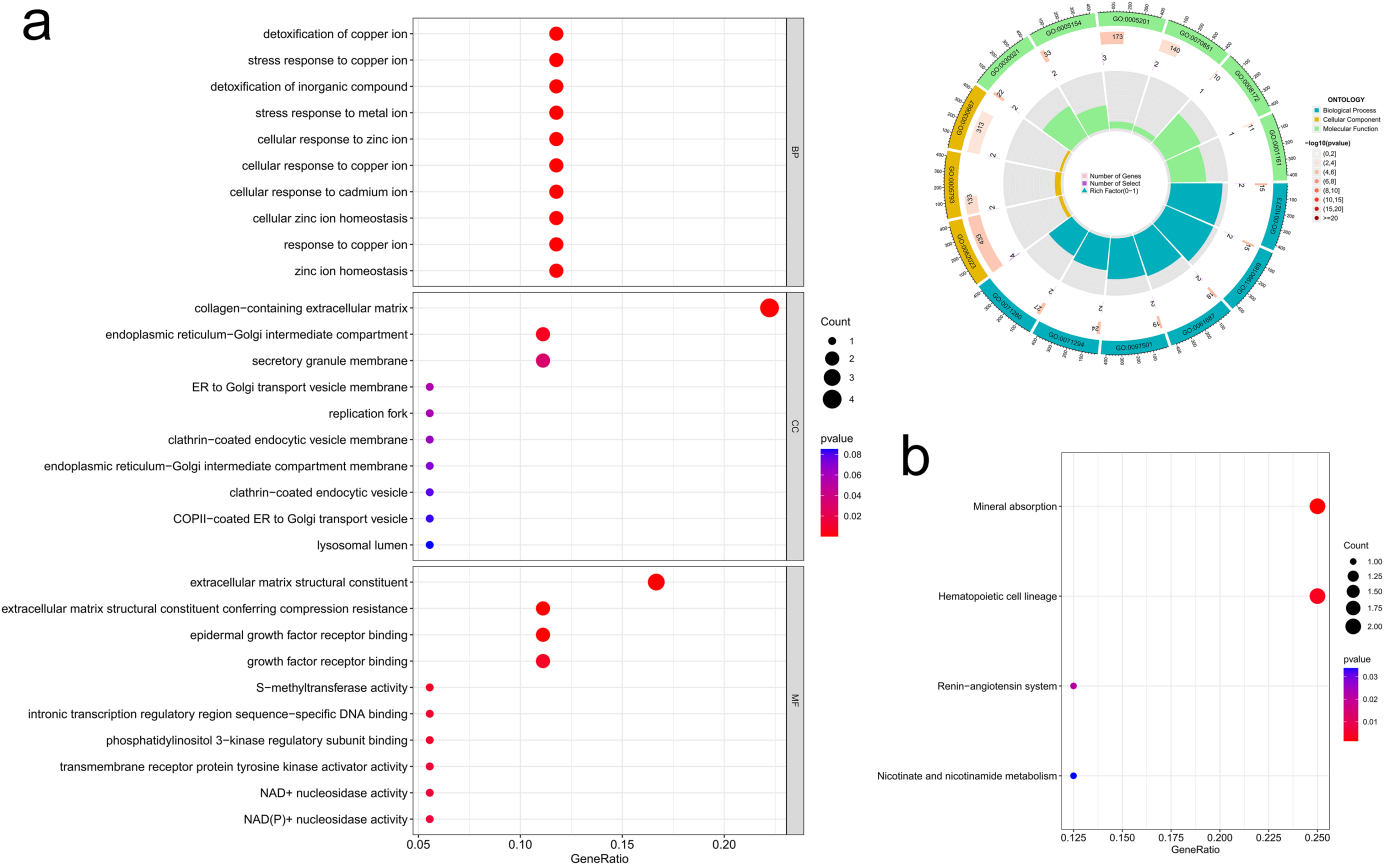
Common DEGs enrichment analysis results. **(a)** Enrichment analysis results of GO (bubble diagram and loop graph); **(b)** enrichment analysis results of KEGG pathways (bubble diagram).

We then examined the potential biological functions of common DEGs using R. GO analysis showed that these common DEGs were significantly enriched in the detoxification of copper ion and inorganic compound, response to metal ion (as part of the biological process), collagen-containing extracellular matrix (ECM) and endoplasmic reticulum-Golgi intermediate compartment (within the cellular component), and ECM structural constituent and growth factor receptor binding (pertaining to molecular function) (Figure 4a). Subsequently, KEGG pathway enrichment analysis revealed that these genes were predominantly clustered in pathways such as mineral absorption, hematopoietic cell lineage, the renin-angiotensin system, and nicotinate and nicotinamide metabolism (Figure 4b). The identification of these enrichment pathways suggests potential mechanistic links to IHF and AF pathology.

### 3.4 PPI network and hub genes analysis

**Figure 5:**
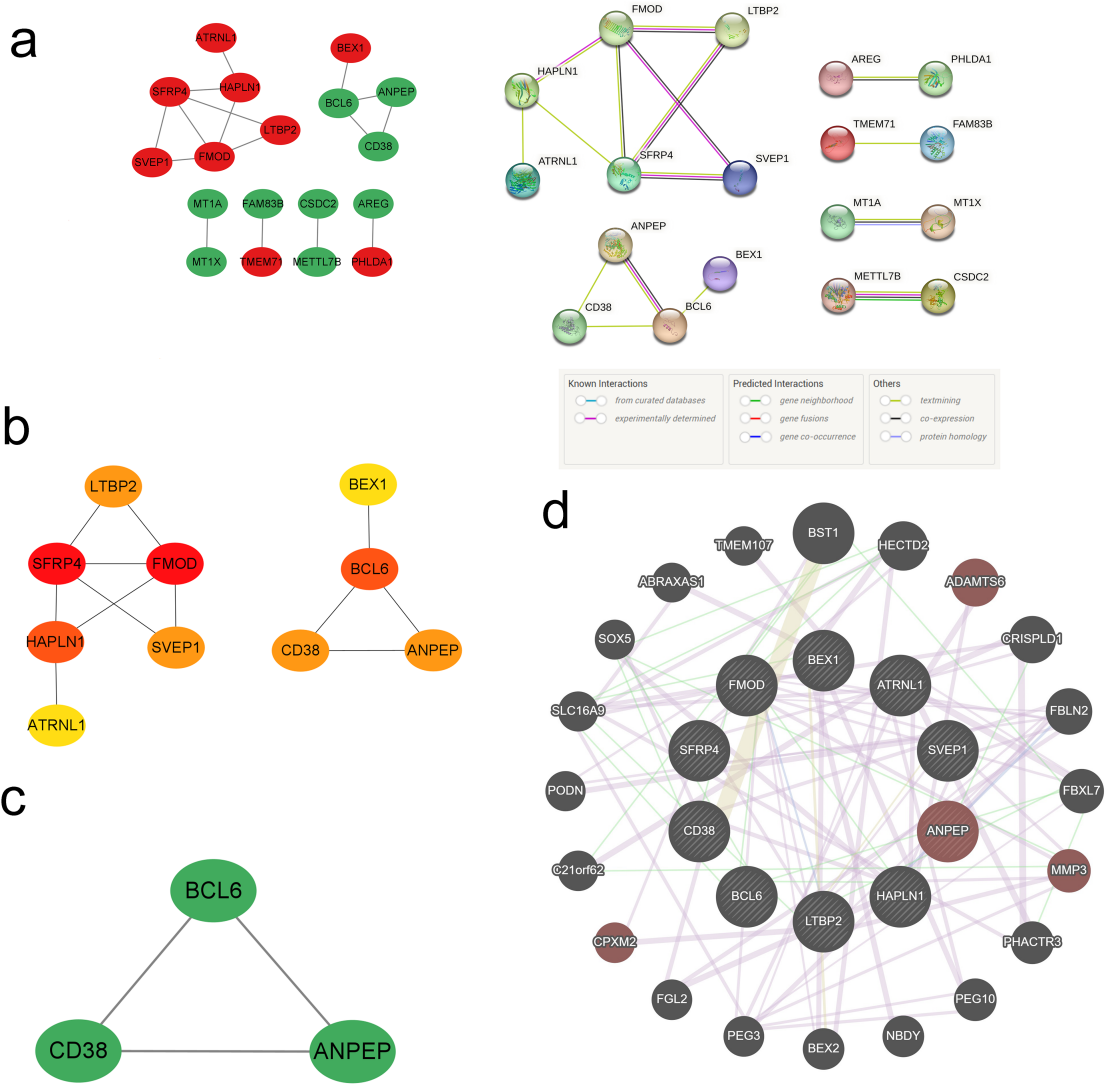
PPI network and hub genes. **(a)** PPI network diagram; **(b)** cytoHubba screening of hub genes; **(c)** MCODE components; **(d)** circular diagram of hub genes from GeneMANIA.

We conducted a PPI network analysis of common DEGs to explore potential interactions among them, using the tools STRING and Cytoscape. Initially, the STRING analysis revealed 20 nodes and 16 edges for the common DEGs, and the value of the network was set to an interaction score >0.15 (Figure 5a). Then, these 20 common DEGs were utilized to construct a network graph in Cytoscape. Within this network, top 10 hub genes were identified using the cytoHubba plugin, including 7 up-regulated genes (SFRP4, FMOD, HAPLN1, LTBP2, SVEP1, ATRNL1, BEX1) and 3 down-regulated genes (BCL6, ANPEP, CD38) (Figure 5b). Subnetworks containing these 3 down-regulated genes were further identified using MCODE (Figure 5c). The GeneMANIA database was subsequently applied to reveal the co-expression networks of the aforementioned genes and to describe their related functions (Figure 5d). Analysis through the UniProt database revealed that SFRP4 primarily participates in the Wnt signaling pathway, while FMOD and LTBP2 contribute to collagen fiber formation and elastic fiber composition, respectively. CD38 was found to be involved in the synthesis of NAADP, a calcium mobilizing agent. These pathways play critical roles in atrial remodeling, myocardial fibrosis, and calcium homeostasis, potentially serving as shared mechanisms and therapeutic targets for patients with IHF and AF.

### 3.5 Enrichment pathways and analysis for hub genes

**Figure 6:**
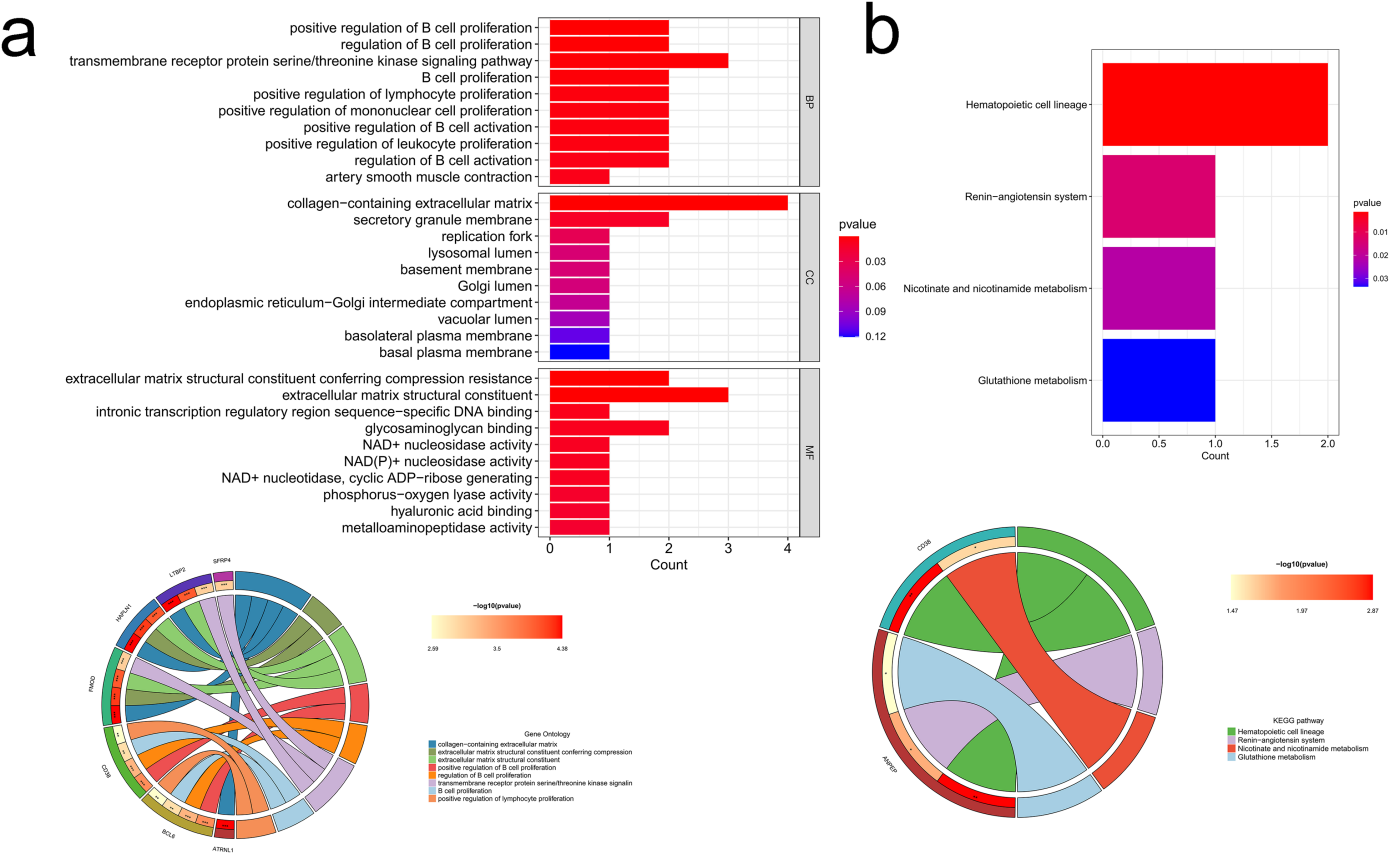
Enrichment analysis results of hub genes. **(a)** GO enrichment analysis of the hub genes; **(b)** KEGG enrichment analysis of the hub genes.

To delve into the biological processes and potential functions associated with the 10 hub genes, we conducted GO and KEGG analysis, and the results were presented in a circos plot. The GO functional enrichment analysis pinpointed that these hub genes were mainly involved in pathways such as collagen-containing ECM, ECM structural constituent, transmembrane receptor protein serine/threonine kinase signaling pathway, B cell proliferation, and positive regulation of lymphocyte proliferation (Figure 6a). Additionally, KEGG analysis highlighted the most enriched pathways, including hematopoietic cell lineage, renin-angiotensin system, nicotinate and nicotinamide metabolism, and glutathione metabolism (Figure 6b).

### 3.6 Validation of hub genes expression

**Figure 7:**
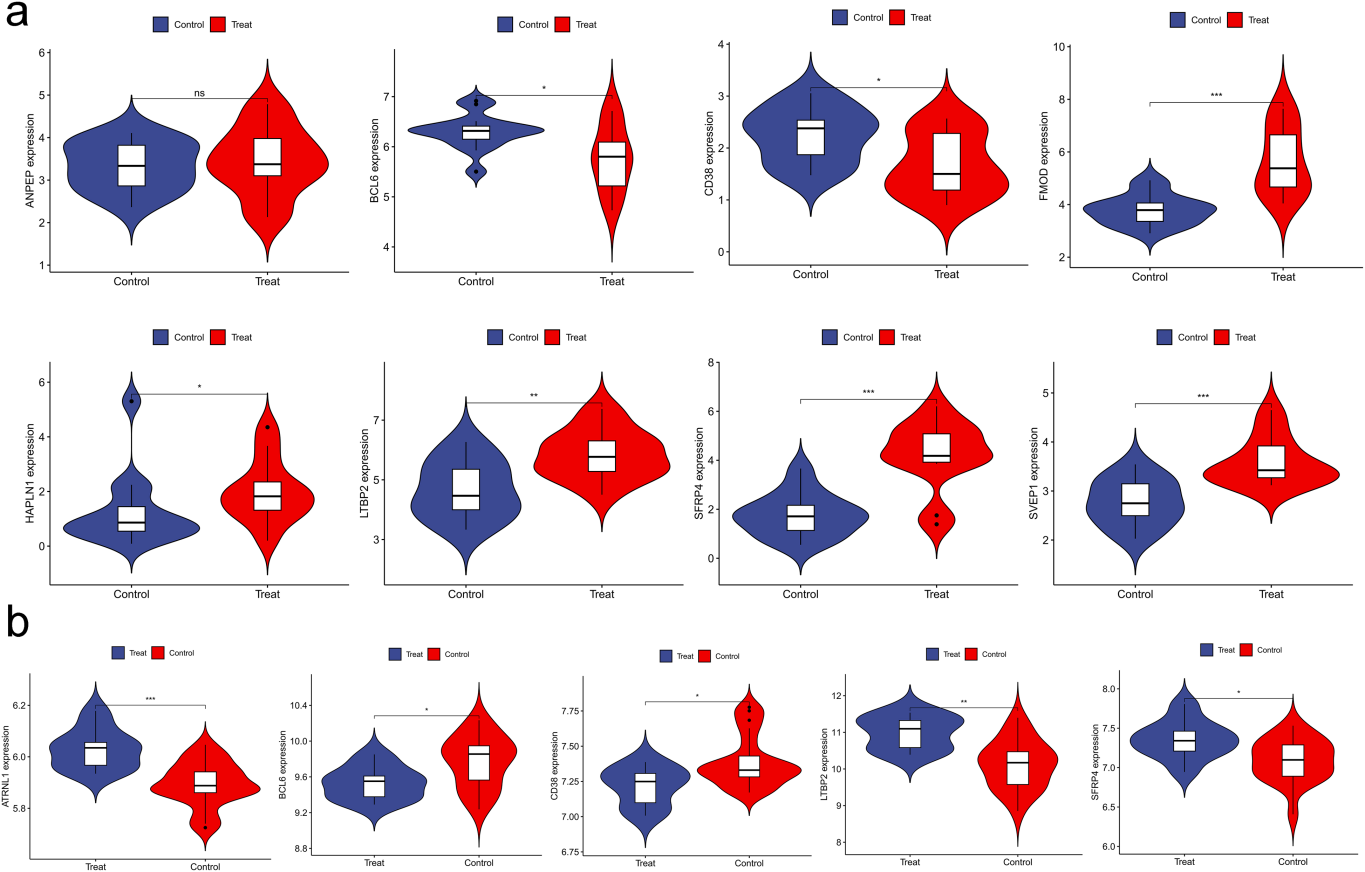
Validation of hub genes expression. **(a)** violin plot of ANPEP, BCL6, CD38, FMOD, HAPLN1, LTBP2, SFRP4, SVEP1; **(b)** violin plot of ATRNL1, BCL6, CD38, LTBP2, SFRP4.

The expression levels of the hub genes identified in the previous biological analysis were validated using the GSE116250 dataset for HF and the GSE2240 dataset for AF from the GEO database. Specifically, among the 10 hub genes, 8 (SFRP4, FMOD, HAPLN1, LTBP2, SVEP1, BCL6, CD38, ATRNL1) were found to have significantly higher expression levels in HF patients compared to non-HF subjects (*P* < 0.05) in the GSE116250 dataset. Furthermore, the expression levels of 5 genes (SFRP4, LTBP2, BCL6, CD38, ATRNL1) were significantly higher in AF patients than in SR patients (*P* < 0.05) in the GSE2240 dataset. These findings further validated our results, as illustrated in Figure 7.

### 3.7 Single-cell analysis of hub gene locations

**Figure 8:**
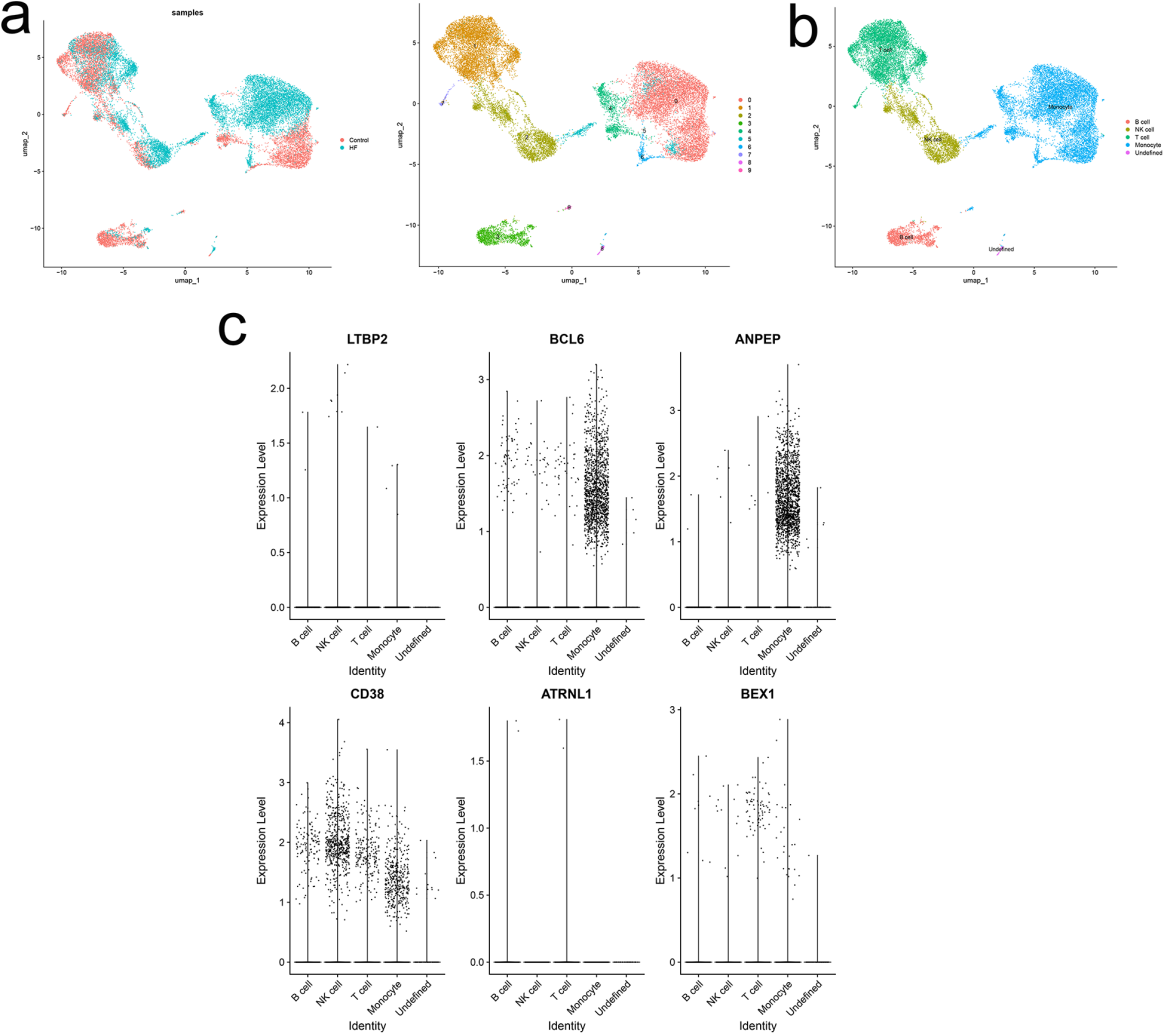
Single-cell analysis of expression profiles of hub genes. **(a)** UMAP of 10 circulating cells in HF. **(b)** UMAP of immune cells in HF. **(c)** Localization analysis of hub genes.

Quality control of two samples from the HF dataset resulted in the identification of 10 distinct cell clusters (Figure 8a). Cell population annotation followed established marker protocols. T cell identification utilized markers CD3E, IL7R, CCR7, CD4, CD8A, and CCL5. NK cell characterization employed KLRB1, NKG7, and GNLY markers. Monocyte populations were defined by LYZ, CD14, CD68, S100A9, FCGR3A, and CD1C expression. B cell identification relied on MS4A1, CD19, and CD79A markers. The analysis yielded five distinct cell populations, including one undefined population (Figure 8b). Localization analysis of key diagnostic markers revealed high expression of BCL6 and ANPEP in monocytes, while CD38 showed high expression in both NK cells and monocytes (Figure 8c). The findings suggested that immune cells significantly influence the HF microenvironment, particularly through monocyte-driven inflammation as a primary driver of HF fibrotic changes. Given that atrial fibrosis is a hallmark of structural cardiac remodeling in AF, monocytes may play a crucial role in both IHF and AF pathogenesis.

## 4 Discussion

AF and HF commonly coexist, forming a complex interplay. The abnormal pressure within the left ventricle in HF patients leads to an escalation in left atrial pressure. Simultaneously, the irregular heartbeat and amplified ventricular rate induced by AF culminate in diminished left ventricular filling time, consequently elevating left atrial pressure (13, 14). During systole, the absence of atrial contraction undermines ventricular filling and compromises diastolic function. With a relentless ascent of ventricular and atrial pressures, cardiac remodeling occurs, resulting in the degeneration of both AF and HF (15). Nonetheless, the precise biological mechanisms interlinking AF and HF remain elusive, especially in ISCH. A comprehensive analysis of the interrelation between these two diseases is thus essential. The identification of associated biomarkers stands as a critical milestone for the development of therapeutic and preventive strategies.

The objective of this study was to elucidate the intrinsic mechanisms and relationships of concurrent IHF and AF, by employing microarray data analysis. Twenty common DEGs were discerned in the two diseases, encompassing 10 hub genes such as SFRP4, FMOD, HAPLN1, LTBP2, SVEP1, BCL6, ANPEP, CD38, ATRNL1, and BEX1. Enrichment analysis revealed that these hub genes were enriched in pathways such as ECM, endoplasmic reticulum−Golgi, response to metal ions, growth factor receptor binding, mineral absorption, hematopoietic cell lineage, renin−angiotensin system, and nicotinate and nicotinamide metabolism. The present study, through hub genes enrichment analysis, unveiled shared signaling pathways between IHF and AF, furnishing novel evidence for their connection. These hub genes were validated in two other datasets. Ultimately, 5 genes were found to be common and statistically significant between IHF and AF, potentially providing insights into the coexistence mechanism of IHF and AF. Moreover, alterations in the expression of FMOD, HAPLN1, and SVEP1 may act as a catalyst for the progression of IHF.

The cardiovascular integrity of healthy adults hinges on Wnt signaling fidelity. Aberrant activation of the Wnt/β-catenin signaling pathway has been shown to correlate with atrial remodeling and regulate cardiac fibroblasts and the ECM, emerging as a significant factor in the development and progression of HF and AF (16–18). SFRP4, secreted frizzled-related protein 4, is a member of the SFRP family. It is associated with G-protein-coupled receptor activity and functions as a soluble modulator within Wnt signaling (19). SFRP4, boasting the highest expression in the heart (20), correlates with the expression of apoptosis-related genes. SFRP4 was historically reported as a transcriptional regulator for HF (21). In cases of HF induced by DCM or coronary artery diseases, pro-apoptotic mRNA levels of SFRP4 experience an upsurge (21, 22). Furthermore, downstream genes (MYH6, MYH7, TNNT2, NKX2-5, and CCND1) regulated by SFRP4 partake in ICM-related diseases like HF and arrhythmias (23). Some studies have demonstrated that knocking out SFRP4 can appreciably mitigate cardiac damage after ischemia-reperfusion injury (22, 24). In our study, SFRP4 expression was upregulated in the cardiac tissue of IHF and AF patients and was a core gene in the PPI, related to 4 key hub genes (FMOD, HAPLN1, LTBP2, SVEP1). These findings highlight its potential role in the pathological processes of IHF and AF, possibly mediated through the Wnt/β-catenin signaling pathway.

FMOD, as a collagen-binding keratan sulfate small leucine-rich proteoglycan, is distinctly expressed within connective tissues and cartilage. It plays an essential role in regulating collagen fiber formation and significantly influences collagen crosslinking. Studies have elucidated that FMOD demonstrates anti-fibrotic properties, adeptly modulating fibrotic responses via interaction with Transforming Growth Factor Beta (TGF-β) (25). An escalation in FMOD levels leads to a consequent reduction in the migration of Cardiac Fibroblasts (CFB) (26). The proliferation, migration, activation of CFB, and excessive secretion of ECM proteins are key to cardiac fibrosis (27), resulting in scar tissue formation, arrhythmia onset, cardiac stiffening, and ultimately leading to HF.

LTBP2, a constituent of the Latent TGF-β1 Binding Protein family, is actively engaged in TGF-β1 signaling regulation and stands as an ECM protein symptomatic of fibrosis. Empirical evidence points out a heightened LTBP2 expression within the fibrotic regions of cardiac tissues in IHF patients, potentially signifying the progression of cardiac fibrosis (28–30). As an atrium-concentrated gene, LTBP2 might serve as a prognostic tool to identify patients susceptible to AF (31).

BCL6, a transcriptional repressor imbued with anti-apoptotic and oncogenic attributes, was initially identified as an oncogene within Non-Hodgkin's B-Cell Lymphoma (32). Recent research has unveiled a contributory role of BCL6 in fostering cardiac fibrosis through the TGF-β1/Smads pathway, in addition to safeguarding against ischemic myocardial injury (33, 34). Furthermore, animal investigations have revealed SVEP1 as an ECM protein expressed in vascular smooth muscle cells. It promotes inflammation and atherosclerosis via integrin, notch, and fibroblast growth factor receptor signaling (35). A recent investigation has identified SVEP1 as a groundbreaking biomarker for HF. The baseline serum concentrations of SVEP1 are as strongly correlated with HF hospitalization or cardiovascular mortality risk as NT-proBNP, and it is independent of other clinical risk factors (36).

CD38, identified as a primary nicotinamide adenine dinucleotide (NAD) hydrolase, can cleave NAD into the second messenger cADPR, an essential compound for calcium signaling, and subsequently hydrolyze cADPR into ADP-ribose (ADPR) (37). Remarkably, the heart manifests the most elevated NAD levels, positioning itself among the organs requiring the most substantial metabolism. Situated within the cardiac endothelium (38), CD38 plays an indispensable role in maintaining intracellular NAD concentrations. The activation of CD38 is a significant cause of endothelial dysfunction following ischemia (39). In the context of CD38 knockout mice, a marked decrease in the calcium signaling molecule cADPR occurs, substantiating an integral role of CD38 in the Ca^2+^/calmodulin-dependent phosphatase/NFAT signaling pathway (40). Calcium homeostasis abnormalities affect myocardial contraction and electrophysiological stability, leading to HF and AF (41). This study suggests that CD38 is downregulated in IHF and AF patients, and is correlated with BCL6 and ANPEP within the MCODE subnetwork. KEGG pathway enrichment analysis delineates that CD38 is operative within nicotinate and nicotinamide metabolism. NAD is predominantly biosynthesized from niacin, nicotinamide, and nicotinamide nucleosides. This signifies that CD38 might participate in the development of these two diseases, thereby potentially serving as an innovative therapeutic target for IHF and AF patients.

Previous research has tendered ANPEP as a prospective blood marker for doxorubicin-induced HF (42), the induced overexpression of BEX1 in cardiac cells has been found to aggravate stress overload-induced cardiac dysfunction and remodeling (43).

SFRP4, FMOD, LTBP2, SVEP1, HAPLN1, and BEX1 were significantly upregulated in the cardiac tissues of patients with IHF and AF, whereas BCL6, CD38, and ANPEP were notably downregulated. GO enrichment analysis revealed that these hub genes were primarily enriched in the ECM, the endoplasmic reticulum-Golgi apparatus, and responses to metal ions. Previous experimental and clinical data suggest that oxidative stress, calcium overload, myocardial remodeling, inflammatory activation, and myofibroblast activation play critical roles not only in HF (44) but also in atrial ECM remodeling and electrical remodeling, contributing to AF (45). In AF, activation of β-catenin may result from enhanced Akt/GSK-3β/β-catenin signaling, which induces atrial fibrosis (46). These observations are consistent with our findings, suggesting that hub gene-mediated ECM metabolic imbalance leads to ventricular dysfunction and myocardial fibrosis, contributing to the progression of AF and IHF. Thus, regulating the ECM could be a potential strategy for managing the microenvironmental characteristics of IHF and AF. Potential therapeutic interventions might include inhibiting the Wnt/β-catenin signaling pathway or the TGF-β1/Smads signaling pathway, thereby suppressing CFB proliferation, attenuating collagen production, minimizing atrial structural remodeling, and preventing AF-induced cardiac fibrosis and subsequent IHF development. At present, no studies report the roles of these hub genes within IHF and AF. Whether they are mere substitutes for cardiac fibrosis or involved in the pathogenesis of IHF and AF remains unclear. This study pioneers the proposal of their interconnections, positing them as probable shared pathogenic mechanisms and therapeutic targets for IHF and AF patients. However, further research is required for validation.

Single-cell analysis of HF patient datasets in this study revealed five distinct cell populations: B cells, T cells, NK cells, monocytes, and undefined cell groups. Six hub genes exhibited varied expression patterns across immune cells, with predominant expression in monocytes, providing preliminary evidence for a potential link between the comorbidity of IHF and AF and the monocyte immune microenvironment.

Inflammation is a fundamental pathophysiological mechanism in the onset and progression of HF and AF (47). Monocytes are key mediators of this process by releasing chemokines, such as TNF*α* and TGF-β, which exert pro-inflammatory and pro-fibrotic effects (48, 49). Excessive cardiac monocyte recruitment induces myocardial injury and remodeling, propagating an escalating damage-recruitment cycle in HF and AF patients (50). Prior studies have demonstrated increased TGF-β expression in cardiac tissue leukocytes during macrophage/monocyte polarization (50), aligning with our findings of elevated BCL6 and ANPEP in monocytes and high CD38 expression in both NK cells and monocytes. PPI network analysis revealed CD38 downregulation in IHF and AF patients, with correlations to BCL6 and ANPEP in the MCODE subnetwork. BCL6 promotes cardiac fibrosis via the TGF-β/Smads pathway. This fibrotic process, a hallmark of structural cardiac remodeling in AF, involves myocardial inflammation and oxidative stress secondary to inflammatory cell infiltration. The process activates CD38, promoting NADH oxidase release and contributing to IHF and AF pathogenesis. Additional studies are warranted to clarify the mechanisms governing CD38 expression in the myocardium and the potential contribution of monocytes as a source of TGF-β. Further investigation into the role of monocytes and their subsets in IHF-AF comorbidity pathophysiology may reveal novel therapeutic targets.

However, this study has certain limitations. Initially, the exploration is circumscribed to the analysis of accessible data, thus necessitating future corroboration via cellular experiments, animal models, or clinical specimens. Additionally, the inconsistencies in the inclusion criteria for datasets from the GEO database and a predominant lack of corresponding clinical pathological determinants such as ICH type (either preserved or reduced ejection fraction), impede a comprehensive analysis of associations of hub genes with disease inception, progression, and prognosis. Furthermore, this study was only a preliminary single-cell analysis result. More research is needed to confirm the connection between key genes and cells and between cells and diseases. We only used HF single-cell data sets for analysis, and have not yet found suitable AF single-cell data, and the data source is also limited. Therefore, in future studies, we will continue to attach importance to the integration of single-cell genes and collect a large number of data sets to strengthen and improve this research result. As well as, subsequent research needs to simulate preclinical human models with both HF and AF with a larger sample size, and these potential biomarkers need to be validated in a more heterogeneous HF population, considering both reduced and preserved ejection fractions.

This rigorous investigation has unearthed numerous intricate associations between IHF and AF, possibly mediated by hub genes (SFRP4, FMOD, HAPLN1, LTBP2, SVEP1, BCL6, ANPEP, CD38, ATRNL1, BEX1). These hub genes serve as potential biomarkers for the concurrent manifestation of IHF and AF, providing novel insights into their underlying pathogenic mechanisms and therapeutic strategies.

## Data Availability

Publicly available datasets were analyzed in this study. This data can be found in the GEO database (https://www.ncbi.nlm.nih.gov/geo/), accession numbers: GSE57338, GSE116250, GSE128188, GSE2240, GSE267644.

## References

[1] Chugh SS Havmoeller R Narayanan K Singh D Rienstra M Benjamin EJ Worldwide epidemiology of atrial fibrillation: a global burden of disease 2010 study. Circulation. (2014) 129(8):837–47. 10.1161/circulationaha.113.005119 24345399 PMC4151302

[2] Savarese G Becher PM Lund LH Seferovic P Rosano GMC Coats AJS. Global burden of heart failure: a comprehensive and updated review of epidemiology. Cardiovasc Res. (2023) 118(17):3272–87. 10.1093/cvr/cvac013 35150240

[3] Dries DL Exner DV Gersh BJ Domanski MJ Waclawiw MA Stevenson LW. Atrial fibrillation is associated with an increased risk for mortality and heart failure progression in patients with asymptomatic and symptomatic left ventricular systolic dysfunction: a retrospective analysis of the SOLVD trials. Studies of left ventricular dysfunction. J Am Coll Cardiol. (1998) 32(3):695–703. 10.1016/s0735-1097(98)00297-6 9741514

[4] Ruddox V Sandven I Munkhaugen J Skattebu J Edvardsen T Otterstad JE. Atrial fibrillation and the risk for myocardial infarction, all-cause mortality and heart failure: a systematic review and meta-analysis. Eur J Prev Cardiol. (2017) 24(14):1555–66. 10.1177/2047487317715769 28617620 PMC5598874

[5] Liew CC Dzau VJ. Molecular genetics and genomics of heart failure. Nat Rev Genet. (2004) 5(11):811–25. 10.1038/nrg1470 15520791

[6] Kittleson MM Ye SQ Irizarry RA Minhas KM Edness G Conte JV Identification of a gene expression profile that differentiates between ischemic and nonischemic cardiomyopathy. Circulation. (2004) 110(22):3444–51. 10.1161/01.Cir.0000148178.19465.11 15557369

[7] Hannenhalli S Putt ME Gilmore JM Wang J Parmacek MS Epstein JA Transcriptional genomics associates FOX transcription factors with human heart failure. Circulation. (2006) 114(12):1269–76. 10.1161/circulationaha.106.632430 16952980

[8] Raunsø J Pedersen OD Dominguez H Hansen ML Møller JE Kjaergaard J Atrial fibrillation in heart failure is associated with an increased risk of death only in patients with ischaemic heart disease. Eur J Heart Fail. (2010) 12(7):692–7. 10.1093/eurjhf/hfq052 20403817

[9] Mercer BN Koshy A Drozd M Walker AMN Patel PA Kearney L Ischemic heart disease modifies the association of atrial fibrillation with mortality in heart failure with reduced ejection fraction. J Am Heart Assoc. (2018) 7(20):e009770. 10.1161/jaha.118.009770 30371286 PMC6474978

[10] Hong KL Borges J Glover B. Catheter ablation for the management of atrial fibrillation: current technical perspectives. Open Heart. (2020) 7(1):e001207. 10.1136/openhrt-2019-001207 32393656 PMC7223467

[11] Zhuang Y Qiao Z Bi X Han D Jiang Q Zhang Y Screening and bioinformatics analysis of crucial gene of heart failure and atrial fibrillation based on GEO database. Medicina (Kaunas). (2022) 58(10):1319. 10.3390/medicina58101319 36295481 PMC9608246

[12] Huang K Lu J Li Q Wang C Ding S Xu X The role of epicardial adipose tissue-derived proteins in heart failure with preserved ejection fraction and atrial fibrillation: a bioinformatics analysis. J Inflamm Res. (2024) 17:6093–111. 10.2147/jir.S466203 39257896 PMC11385935

[13] Batul SA Gopinathannair R. Atrial fibrillation in heart failure: a therapeutic challenge of our times. Korean Circ J. (2017) 47(5):644–62. 10.4070/kcj.2017.0040 28955382 PMC5614940

[14] Ling LH Kistler PM Kalman JM Schilling RJ Hunter RJ. Comorbidity of atrial fibrillation and heart failure. Nat Rev Cardiol. (2016) 13(3):131–47. 10.1038/nrcardio.2015.191 26658575

[15] Ferreira JP Santos M. Heart failure and atrial fibrillation: from basic science to clinical practice. Int J Mol Sci. (2015) 16(2):3133–47. 10.3390/ijms16023133 25647414 PMC4346884

[16] Foulquier S Daskalopoulos EP Lluri G Hermans KCM Deb A Blankesteijn WM. WNT signaling in cardiac and vascular disease. Pharmacol Rev. (2018) 70(1):68–141. 10.1124/pr.117.013896 29247129 PMC6040091

[17] Wolke C Antileo E Lendeckel U. WNT signaling in atrial fibrillation. Exp Biol Med (Maywood). (2021) 246(9):1112–20. 10.1177/1535370221994086 33641440 PMC8113736

[18] Chacko P Bhuta S Meenakshisundaram C Moustafa A Davis A Gupta R. Prevalence of heart failure with preserved ejection fraction in patients undergoing atrial fibrillation ablation based on resting and post-tachycardia pacing left atrial pressure. Am J Cardiol. (2023) 205:445–50. 10.1016/j.amjcard.2023.07.179 37666016

[19] O'Leary NA Wright MW Brister JR Ciufo S Haddad D McVeigh R Reference sequence (RefSeq) database at NCBI: current status, taxonomic expansion, and functional annotation. Nucleic Acids Res. (2016) 44(D1):D733–45. 10.1093/nar/gkv1189 26553804 PMC4702849

[20] Finch PW He X Kelley MJ Uren A Schaudies RP Popescu NC Purification and molecular cloning of a secreted, frizzled-related antagonist of Wnt action. Proc Natl Acad Sci U S A. (1997) 94(13):6770–5. 10.1073/pnas.94.13.6770 9192640 PMC21233

[21] Schumann H Holtz J Zerkowski HR Hatzfeld M. Expression of secreted frizzled related proteins 3 and 4 in human ventricular myocardium correlates with apoptosis related gene expression. Cardiovasc Res. (2000) 45(3):720–8. 10.1016/s0008-6363(99)00376-4 10728394

[22] Ji Q Zhang J Du Y Zhu E Wang Z Que B Human epicardial adipose tissue-derived and circulating secreted frizzled-related protein 4 (SFRP4) levels are increased in patients with coronary artery disease. Cardiovasc Diabetol. (2017) 16(1):133. 10.1186/s12933-017-0612-9 29037197 PMC5644066

[23] Alimadadi A Aryal S Manandhar I Joe B Cheng X. Identification of upstream transcriptional regulators of ischemic cardiomyopathy using cardiac RNA-seq meta-analysis. Int J Mol Sci. (2020) 21(10):3472. 10.3390/ijms21103472 32423033 PMC7278960

[24] Zeng W Cao Y Jiang W Kang G Huang J Xie S. Knockdown of Sfrp4 attenuates apoptosis to protect against myocardial ischemia/reperfusion injury. J Pharmacol Sci. (2019) 140(1):14–9. 10.1016/j.jphs.2019.04.003 31113729

[25] Zheng Z Nguyen C Zhang X Khorasani H Wang JZ Zara JN Delayed wound closure in fibromodulin-deficient mice is associated with increased TGF-β3 signaling. J Invest Dermatol. (2011) 131(3):769–78. 10.1038/jid.2010.381 21191417 PMC4073663

[26] Andenæs K Lunde IG Mohammadzadeh N Dahl CP Aronsen JM Strand ME The extracellular matrix proteoglycan fibromodulin is upregulated in clinical and experimental heart failure and affects cardiac remodeling. PLoS One. (2018) 13(7):e0201422. 10.1371/journal.pone.0201422 30052659 PMC6063439

[27] Travers JG Kamal FA Robbins J Yutzey KE Blaxall BC. Cardiac fibrosis: the fibroblast awakens. Circ Res. (2016) 118(6):1021–40. 10.1161/circresaha.115.306565 26987915 PMC4800485

[28] Park S Ranjbarvaziri S Lay FD Zhao P Miller MJ Dhaliwal JS Genetic regulation of fibroblast activation and proliferation in cardiac fibrosis. Circulation. (2018) 138(12):1224–35. 10.1161/circulationaha.118.035420 29950403 PMC6202226

[29] Park S Ranjbarvaziri S Zhao P Ardehali R. Cardiac fibrosis is associated with decreased circulating levels of full-length CILP in heart failure. JACC Basic Trans Sci. (2020) 5(5):432–43. 10.1016/j.jacbts.2020.01.016 PMC7251193 32478206

[30] Yang KC Yamada KA Patel AY Topkara VK George I Cheema FH Deep RNA sequencing reveals dynamic regulation of myocardial noncoding RNAs in failing human heart and remodeling with mechanical circulatory support. Circulation. (2014) 129(9):1009–21. 10.1161/circulationaha.113.003863 24429688 PMC3967509

[31] Maass AH De Jong AM Smit MD Gouweleeuw L de Boer RA Van Gilst WH Cardiac gene expression profiling—the quest for an atrium-specific biomarker. Neth Heart J. (2010) 18(12):610–4. 10.1007/s12471-010-0844-8 21301625 PMC3018608

[32] Ohno H. Pathogenetic and clinical implications of non-immunoglobulin; BCL6 translocations in B-cell non-Hodgkin’s lymphoma. J Clin Exp Hematopathol. (2006) 46(2):43–53. 10.3960/jslrt.46.43 17142954

[33] Lin JM Hsu CH Chen JC Kao SH Lin YC. BCL-6 promotes the methylation of miR-34a by recruiting EZH2 and upregulating CTRP9 to protect ischemic myocardial injury. BioFactors (Oxford, England). (2021) 47(3):386–402. 10.1002/biof.1704 33502806

[34] Li PF He RH Shi SB Li R Wang QT Rao GT Modulation of miR-10a-mediated TGF-β1/Smads signaling affects atrial fibrillation-induced cardiac fibrosis and cardiac fibroblast proliferation. Biosci Rep. (2019) 39(2):BSR20181931. 10.1042/bsr20181931 30683806 PMC6367129

[35] Jung IH Elenbaas JS Alisio A Santana K Young EP Kang CJ SVEP1 Is a human coronary artery disease locus that promotes atherosclerosis. Sci Transl Med. (2021) 13(586):eabe0357. 10.1126/scitranslmed.abe0357 33762433 PMC8109261

[36] Zhang L Cunningham JW Claggett BL Jacob J Mendelson MM Serrano-Fernandez P Aptamer proteomics for biomarker discovery in heart failure with reduced ejection fraction. Circulation. (2022) 146(18):1411–4. 10.1161/circulationaha.122.061481 36029463

[37] Gul R Park DR Shawl AI Im SY Nam TS Lee SH Nicotinic acid adenine dinucleotide phosphate (NAADP) and cyclic ADP-ribose (cADPR) mediate Ca2+ signaling in cardiac hypertrophy induced by β-adrenergic stimulation. PLoS One. (2016) 11(3):e0149125. 10.1371/journal.pone.0149125 26959359 PMC4784992

[38] Reyes LA Boslett J Varadharaj S De Pascali F Hemann C Druhan LJ Depletion of NADP(H) due to CD38 activation triggers endothelial dysfunction in the postischemic heart. Proc Natl Acad Sci U S A. (2015) 112(37):11648–53. 10.1073/pnas.1505556112 26297248 PMC4577172

[39] Tannous C Booz GW Altara R Muhieddine DH Mericskay M Refaat MM Nicotinamide adenine dinucleotide: biosynthesis, consumption and therapeutic role in cardiac diseases. Acta Physiol (Oxford, England). (2021) 231(3):e13551. 10.1111/apha.13551 32853469

[40] Guan XH Hong X Zhao N Liu XH Xiao YF Chen TT CD38 Promotes angiotensin II-induced cardiac hypertrophy. J Cell Mol Med. (2017) 21(8):1492–502. 10.1111/jcmm.13076 28296029 PMC5542907

[41] Ohkusa T Ueyama T Yamada J Yano M Fujumura Y Esato K Alterations in cardiac sarcoplasmic reticulum Ca2+ regulatory proteins in the atrial tissue of patients with chronic atrial fibrillation. J Am Coll Cardiol. (1999) 34(1):255–63. 10.1016/s0735-1097(99)00169-2 10400019

[42] Wan GX Ji LH Xia WB Cheng L Zhang YG. Bioinformatics identification of potential candidate blood indicators for doxorubicin-induced heart failure. Exp Ther Med. (2018) 16(3):2534–44. 10.3892/etm.2018.6482 30186487 PMC6122467

[43] Accornero F Schips TG Petrosino JM Gu SQ Kanisicak O van Berlo JH BEX1 Is an RNA-dependent mediator of cardiomyopathy. Nat Commun. (2017) 8(1):1875. 10.1038/s41467-017-02005-1 29192139 PMC5709413

[44] Kemp CD Conte JV. The pathophysiology of heart failure. Cardiovasc Pathol. (2012) 21(5):365–71. 10.1016/j.carpath.2011.11.007 22227365

[45] Corradi D. Atrial fibrillation from the pathologist’s perspective. Cardiovasc Pathol. (2014) 23(2):71–84. 10.1016/j.carpath.2013.12.001 24462196

[46] Lin R Wu S Zhu D Qin M Liu X. Osteopontin induces atrial fibrosis by activating akt/GSK-3β/β-catenin pathway and suppressing autophagy. Life Sci. (2020) 245:117328. 10.1016/j.lfs.2020.117328 31954162

[47] Naser JA Lee E Scott CG Kennedy AM Pellikka PA Lin G Prevalence and incidence of diastolic dysfunction in atrial fibrillation: clinical implications. Eur Heart J. (2023) 44(48):5049–60. 10.1093/eurheartj/ehad592 37639219 PMC13386646

[48] Armstrong EJ Morrow DA Sabatine MS. Inflammatory biomarkers in acute coronary syndromes: part I: introduction and cytokines. Circulation. (2006) 113(6):e72–5. 10.1161/CIRCULATIONAHA.105.595520 16476853

[49] Seta Y Shan K Bozkurt B Oral H Mann DL. Basic mechanisms in heart failure: the cytokine hypothesis. J Card Fail. (1996) 2(3):243–9. 10.1016/s1071-9164(96)80047-9 8891862

[50] Shahid F Lip GYH Shantsila E. Role of monocytes in heart failure and atrial fibrillation. J Am Heart Assoc. (2018) 7(3):e007849. 10.1161/JAHA.117.007849 29419389 PMC5850261

